# Clinical and genetic analysis *VSX1* variants among families with keratoconus in northwest China

**DOI:** 10.3389/fgene.2023.1145426

**Published:** 2023-03-14

**Authors:** Jinjin Zhang, Bo Cai, Limei Ma, Yixuan Qin, Shuai Li, Caihong Sun, Jing Liang, Yu Han, Wenjuan Zhuang

**Affiliations:** ^1^ Third Clinical Medical College of Ningxia Medical University, People’s Hospital of Ningxia Hui Autonomous Region, Yinchuan, China; ^2^ Ningxia Eye Hospital, People’s Hospital of Ningxia Hui Autonomous Region, Yinchuan, China; ^3^ College of Medical Technology, North Minzu University, Yinchuan, China

**Keywords:** keratoconus (KC), gene, *VSX1*, variants, topographic, biomechanical

## Abstract

**Purpose:** To screen *VSX1* gene sequence variations and describe the clinical features of families with keratoconus (KC) from northwest China.

**Methods:** We screened *VSX1* sequence variations and clinical data of 37 families including 37 probands with diagnosed KC from Ningxia Eye Hospital (China). *VSX1* was screened by targeted next-generation sequencing (NGS) and verified by Sanger sequencing. In silico analysis including Mutation Taster, MutationAssessor, PROVEAN, MetaLR, FATHMM, M-CAP, FATHMM-XF_coding and DANN was performed to identify the pathogenicity of the sequence variations as well as the conserved amino acid variations of *VSX1* was implemented by Clustal X. All subjects were assessed in Pentacam Scheimpflug tomography and corneal biomechanical Corvis ST examinations.

**Results:** Five *VSX1* gene variants, were identified in six (16.2%) unrelated families with KC. In silico analysis predicted deleterious effects of the three missense variants (p.G342E, p.G160V, and p.L17V) in the encoded protein. Another previously reported synonymous variation (p.R27R) in the first exon and one heterozygous change in the first intron (c.425-73C>T) were identified in three KC families. Clinical examination of the asymptomatic first-degree parents from these six families who shared the gene with the proband had suspected KC changes in topographic and biomechanical markers. These variants co-segregated with the disease phenotype in all affected individuals but not in unaffected family members or healthy controls, though with variable expressivity.

**Conclusion:** The variant p.G342E of *VSX1* is implicated in the pathogenesis of KC, which expands the range of the spectrum of *VSX1* mutations with an autosomal dominant inheritance pattern and variable expression in the clinical phenotype. Genetic screening combined with clinical phenotype may help in the genetic counseling of patients with KC and identification of individuals with subclinical KC.

## 1 Introduction

Keratoconus (KC) is a degenerative disease caused by progressive thinning and ectasia of the bilateral cornea, resulting in irregular astigmatism, reduced vision and even blindness (Chen et al., 2021). The prevalence of KC worldwide varies greatly in different regions and ethnicities, ranging from 0.05% to 0.23% (Shi, 2018). Furthermore, the prevalence of KC in Asians is 4.4 times greater than that in Caucasians, ranging from 0.9% to 3.3% in the Middle East and Asia, respectively (Lucas and Burdon, 2020). However, the prevalence of KC is likely to be underestimated since most studies have been conducted in hospitals and clinics among patients who are usually symptomatic and so early diagnosis is missed. Hence, although large epidemiological studies have been lacking in China (Chinese Medical Association Ophthalmology Branch Corneal Disease Group, 2019), we speculate that the prevalence of KC in Ningxia may be much higher than reported due to the effects of the dry environment and prolonged ultraviolet radiation exposure (Sugar and Macsai, 2012).

Over the years, many studies have confirmed that both external environment factors and genetic variants contribute to the disease (Gomes et al., 2015). Genes reported to be related to KC include *LOX* (5q23.2), *COL5A1* (9q34.2-.3), *CAST* (5q15), *RAB3GAP1* (2q21.3), *HGF* (7q21.1), *FNDC3B* (3q26.31), *FOXO1* (13q14.1), *PNPLA2* (11p15.5), *MAML2* (11q21), *TGFBI* (5q31.1), *DOCK9* (13q32.3), *MPDZ*/*NF1B* (9p23), *WNT10A* (2q35), *ZEB1* (10p11.2), *SOD1* (21q22.1), *IMMP2L* (7q31.1), COL4A3 (2q36.3), *COL4A4* (2q36.3), *VSX1* (20p11.2), *TSC1* (9q34), *IL1A* (2q13), *TIMP3* (22q12.3), *IL1B* (2q13), *ZNF469* (16q24.2), and *PPIP5K2* (5q21.2) (Bykhovskaya and Rabinowitz, 2021), while *VSX1* is the most extensively studied and the only candidate gene so far confirmed to be associated with KC (Bykhovskaya and Rabinowitz, 2021). Although the role of *VSX1* in the pathogenesis of KC is not entirely clear, studies in different regions have confirmed its association with KC and hypothesized that *VSX1* variants play an important role in the development of this condition (Barbaro et al., 2006; Wang et al., 2019; Chen et al., 2021). However, while the majority of previous studies on KC in different regions have been conducted mainly on sporadic patients, reports of studies on pedigrees are rare (Mok et al., 2008; Jeoung et al., 2012; Guan et al., 2017). It has been established that 6%–8% of first-degree relatives in KC families will have the disease, and are at a higher risk than the general population (Chang and Chodosh, 2013; Chen et al., 2021). Therefore, pedigree studies may provide a better understanding of the pathological mechanism of KC. In this study, we conducted both *VSX1* variant screening and clinical examinations of families affected by KC to explore the potential of molecular screening as an approach to detecting subclinical or suspected KC at an extremely early stage before the emergence of clinical signs.

## 2 Materials and methods

### 2.1 Patient information

This descriptive cross-sectional study was approved by the Institutional Review Board of the Ningxia Hui Autonomous Region People’s Hospital (China). The study adhered to the tenets of the Declaration of Helsinki and informed consent was obtained from all subjects. A total of 37 unrelated families were enrolled from the Ningxia Eye Hospital, including a proband with clinically diagnosed KC and their first-degree relatives, regardless of whether they were asymptomatic or diagnosed as KC. In addition, 56 ethnically- and age-matched healthy people without other ocular diseases were enrolled as the control group [no obvious eye disease, normal corneal topography (Pentacam-HR), myopia > −3.0D].

All patients were diagnosed with KC according to the following criteria: 1) one of the following corneal features: thin corneal stroma, Vogt striae, Fleischer ring, Munson sign; 2) inferior-superior (I-S) index >1.5, Kmax (maximum keratometry) >47 D, and the difference in Kmax between the two eyes >1 D; and 3) abnormal corneal topography: asymmetric bow tie, central or inferior steepening (Chen et al., 2021).

### 2.2 Clinical examinations

The following clinical examinations were conducted: slit lamp microscopy, intraocular pressure (IOP), best corrected visual acuity (BCVA), Pentacam Scheimpflug tomography, corneal biomechanics (Corvis ST). Eleven parameters from Pentacam [max keratometry (Kmax), total deviation value (BAD-D), front elevation at the corneal thinnest location (F.Ele.Th), back elevation at the corneal thinnest location (B.Ele.Th), thinnest pachymetry (TP), deviation of front elevation difference map (Df), deviation of back elevation difference map (Db), deviation of average pachymetric progression (Dp), deviation of minimum thickness (Dt), deviation of Ambrosio relational thickness maximum (Da), Ambrosio relational thickness maximum (ARTmax)] and two parameters with sensitivity for diagnosis of KC from Corvis ST [Corvis biomechanical index (CBI), tomographic and biomechanical index (TBI)] were collected. The differences among the clinical data of the proband and first-degree relatives, especially asymptomatic sequence variation carriers, were analyzed. For the Pentacam Scheimpflug tomography and Corvis ST examinations, the yellow region highlighted represents suspicious parameters, while the red region highlighted implies abnormal parameters.

### 2.3 *VSX1* sequence variation screening

For each subject, peripheral venous blood was collected into EDTA anticoagulation tubes. Genomic DNA was extracted using the TIANamp Blood DNA Kit (TIANGEN, Beijing, China) according to the manufacturer’s instructions. For *VSX1* sequence variation screening, the extracted DNA was purified and cleaved, followed by terminal repair, 3′-end adenylation, size selection, PCR amplification, exome library hybridization, library cleaning and purification (TIANSeq, TIANGEN, Beijing, China) according to the manufacturer’s instructions. After PCR amplification, the *VSX1* sequence was sequenced in the target region using the Illumina (San Diego, CA, United States) sequencing platform. The sequence data were mapped to the reference human genome 19.

### 2.4 Bioinformatic analysis

To predict the pathogenicity of *VSX1* variants, we employed a series of *in silico* prediction tools, including Mutation Taster (http://www.mutationtaster.org), MutationAssessor (http://mutationassessor.org/), PROVEAN, MetaLR, FATHMM, FATHMM-XF_coding, DANN (https://grch37.ensembl.org/Tools/VEP) and M-cap, which is the latest online software tool for analyzing and predicting pathogenicity, with a high accuracy rate of 95% and good reliability. The minor allele frequency (MAF) of variants was determined using the 1,000 Genome database (1,000G, http://browser.1000genomes.org), Genome Aggregation Database (gnomAD, https://gnomad.broadinstitute.org/), and 6,500 Exome Sequencing Project database (esp6,500, http://evs.gs.washington.edu/EVS/). Mutations of conserved VSX1 amino acids in different species were predicted using Clustal X and edited by BioEdit. The VSX1 protein structure modeling was performed with SMART (http://smart.embl-heidelberg.de/). Genetic co-segregation was confirmed by Sanger in the families with identified *VSX1* gene variants.

### 2.5 Whole exome sequencing

In this study, patients with *VSX1* sequence variation sites were simultaneously tested by whole exome sequencing (WES) according to a previously described method (Chen et al., 2021), and the relevant pathogenic KC genes (Bykhovskaya and Rabinowitz, 2021) formed the focus of further investigations.

## 3 Results

### 3.1 Study subjects

In this study, we enrolled 37 probands (25 males and 12 females, aged 10–45 years) were diagnosed as having clinical KC. The first-degree relatives without any self-reported complaints or symptoms among the families of the 37 probands were also enrolled.

### 3.2 *VSX1* gene variants

As shown in Table 1, five variants of *VSX1* were detected in six unrelated KC families [1 intron heterozygous variant c.425-73C>T and 4 exon heterozygous variants, c.81C>T (p.R27R), c.479G>T (p.G160V), c.1025G>A (p.G342E), and c.49C>G (p.L17V)]. The c.425-73C>T variant in the first intron was identified in a single individual and was previously unreported. Three missense variants consisting of one p.G342E variant in exon 5 and two p.G160V variants in exon 2 as well as one p.L17V variant in exon 1 were identified in three different individuals. Notably, one previously reported synonymous p.R27R variant in exon 1 was detected in twoKC patients. All of these variants were absent in the control subjects.

**TABLE 1:** Summary of bioinformatics data of *VSX1* sequence variations identified by NGS in the study subjects.

Sequence variation	Genotype	Change site	1,000G	gnomAD	esp6500	Mutation taster	Mutation assessor	FATHMM	PROVEAN	MetaLR	M-CAP	FATHMM-XF_coding	DANN
c.1025G>A (p.G342E)	Het	Exon 5	NI	0.00001	NI	N	L	D	N	D	D	T	0.676
c.479G>T (p.G160V)	Het	Exon 2	0.00259	0.00097	NI	D	M	D	D	D	NI	D	0.974
c.49C>G (p.L17V)	Het	Exon 1	0.00179	0.00076	NI	N	M	D	N	D	NI	T	0.991
c.81C>T (p.R27R)	Het	Exon 1	0.00119	0.00094	NI	NI	NI	NI	NI	NI	NI	NI	NI
c.425-73C>T	Het	Intron 1	0.00019	0.00009	NI	NI	NI	NI	NI	NI	NI	NI	NI

Het, heterozygosis; NI, no information.

Mutation Taster: D: disease-causing, N: probably harmless; Mutation Assessor: H: high; M: medium; L: low; N: neutral; FATHMM: D: deleterious; T: tolerated; PROVEAN: D: deleterious; N: neutral.

MetaLR: D: deleterious, T: tolerated; CAP: D: damaging; T: tolerated; FATHMM-XF_coding: D: deleterious, T: tolerated.

DANN: higher values indicate that the mutation is more deleterious.

**FIGURE 1:**
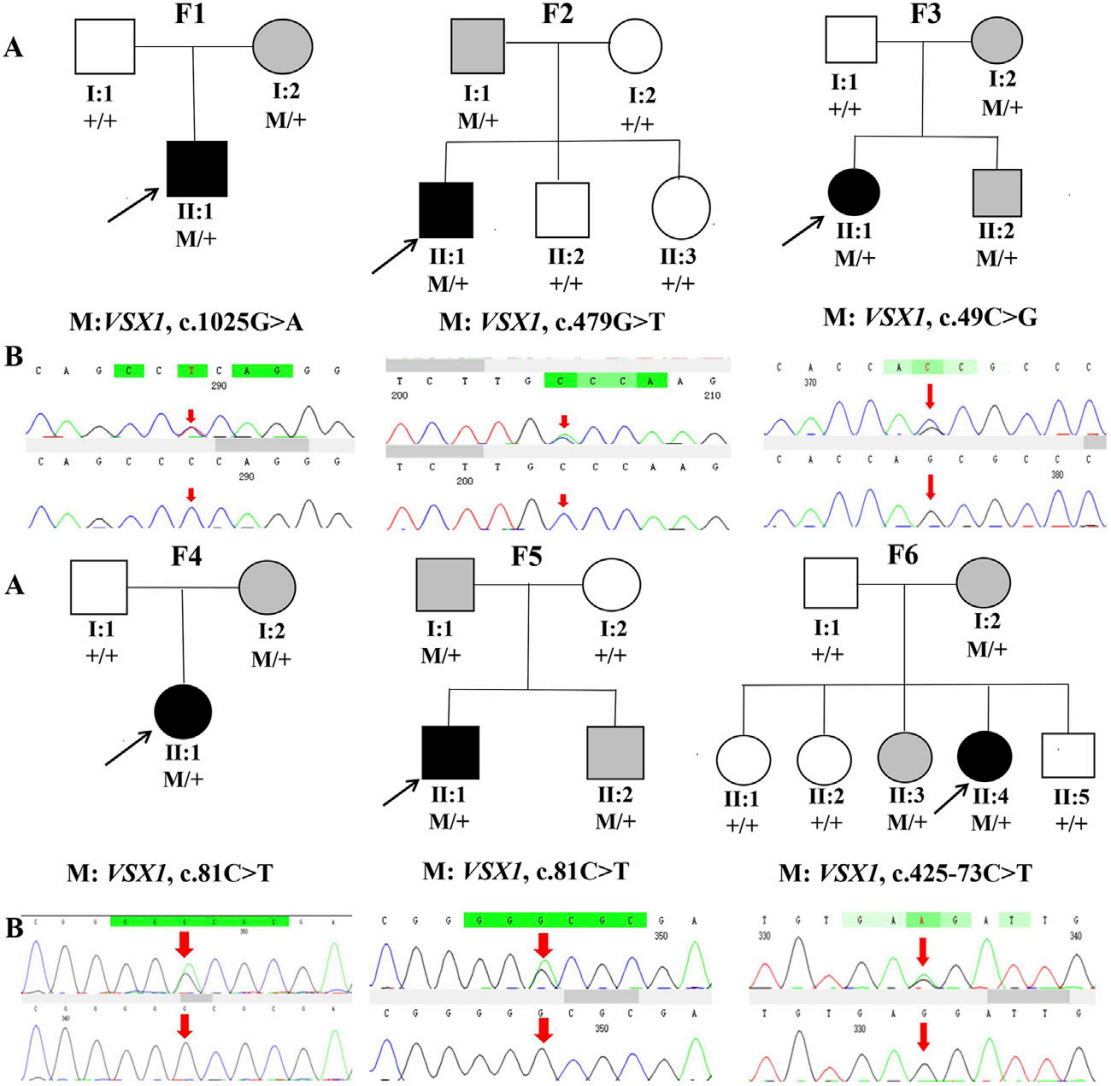
Pedigrees and DNA sequence chromatograms of affected (top) and unaffected (bottom) individuals among the sixKC families. **(A)** Pedigree charts. Black symbols indicate the proband, white symbols indicate unaffected individuals, gray symbol indicate asymptomatic variant carriers with suspicious clinical parameters in Pentacam and Corvis ST. M represents a variant, and + indicates a normal allele. **(B)** The Sequence chromatograms; R arrow represents a mutant type.

**FIGURE 2:**
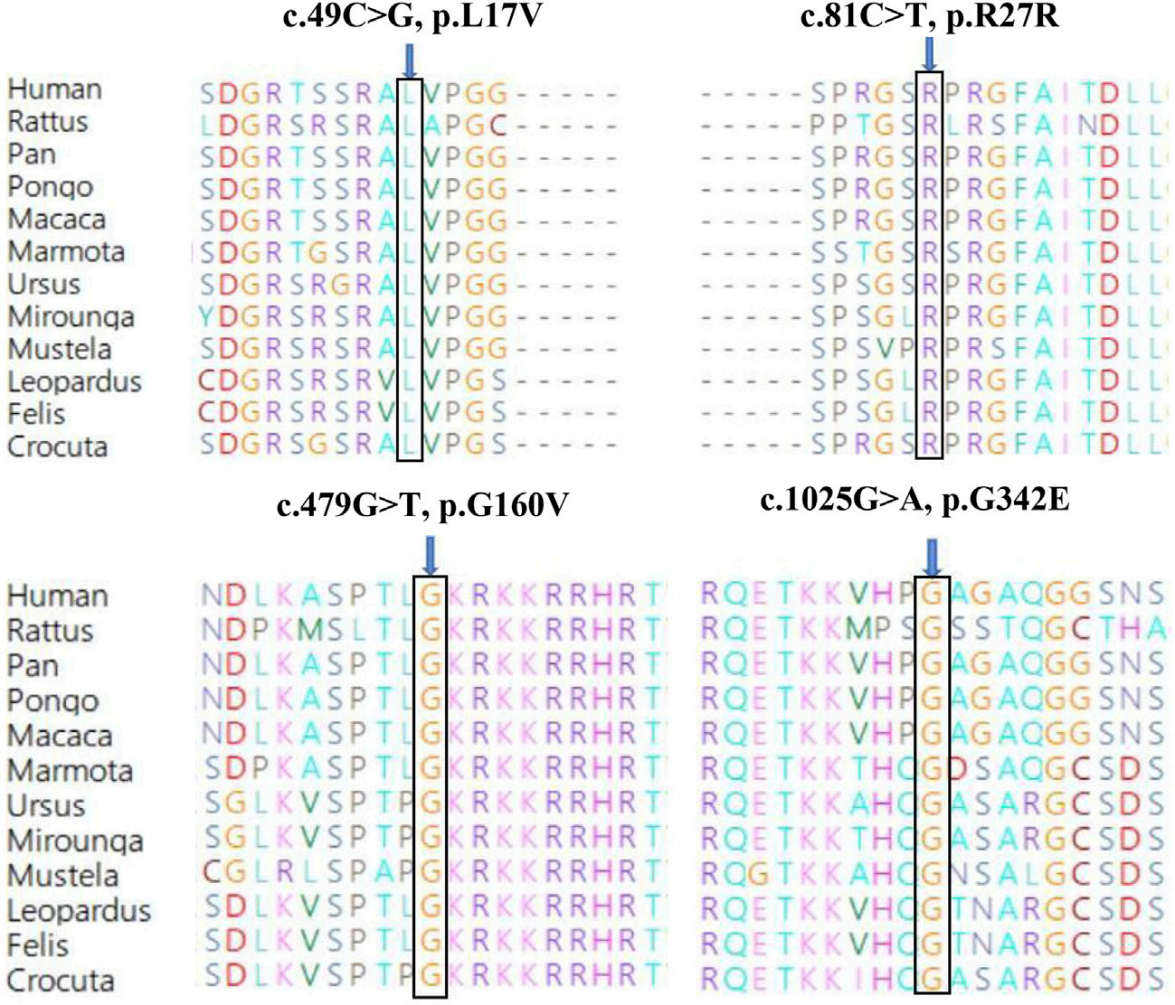
Conservation analysis revealed evolutionary conservation of the *VSX1* variants.Amino acid sequence alignment of the human VSX1 protein with other species. Leu 17, Arg 27, Gly 160 and Gly 342 are shown in blue.

**FIGURE 3:**
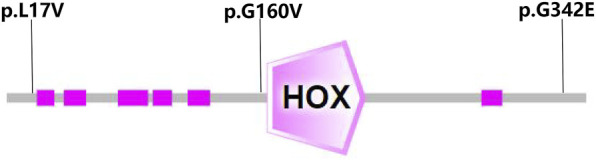
Structure modeling showed that the three missense mutations, p.G160V, p.G342E and p.L17V, were located in the functional domain of the *VSX1* gene.

Genetic co-segregation of the *VSX1* variants identified was confirmed in the families by Sanger sequencing verification (Figure 1). The MAF of each variant was less than 0.005 in the 1,000G, gnomAD and esp6,500 databases. The missense variants, p.G342E, p.G160V, and p.L17V, were predicted to be deleterious by *in silico* tools (Table 1). In addition, all missense variants were highly conserved across different species (Figure 2). SMART protein structure modeling showed that the three missense mutations, p.G342E, p.G160V and p.L17V, were located in the functional domain of *VSX1* (Figure 3).

### 3.3 Whole exome sequencing

Combined analysis of MAF value, pathogenicity analysis, and family co-segregation ruled out the possibility of other keratoconus genes or novel variants, as whole exome sequencing was performed on these KC patients with *VSX1* sequence variation sites.

### 3.4 Clinical data of families with genetic variation

The general clinical data of the probands in the families with *VSX1* variants are shown in Table 2. It is noteworthy that the two individuals with the synonymous p.R27R mutation had identical clinical phenotypes. Both had BCVA of 1.0 and showed high curvature in topographic examinations and no characteristic corneal changes other than thin corneal thickness. Furthermore, Sanger sequencing revealed the same variant in the asymptomatic parents.

**TABLE 2:** The general clinical data of the probands in the six families with *VSX1* sequence variations.

Family ID	sequence variation	Source	Age (years)	Cornea	BCVA (OD; OS)	Kmax (D)	TCT (μm)
						(OD; OS)	(OD; OS)
F1	c.1025G>A (p.G342E)	Mother	19	OU: Fleischer’s ring, Vogt’s line	0.12; 0.12	57.9; 56.7	478; 484
F2	c.479G>T (p.G160V)	Father	12	OD: clear	0.8; 0.02	53.7; 91.8	486; 391
				OS: Fleischer’s ring			
F3	c.49C>G (p.L17V)	Mother	24	OD: Fleischer’s ring	0.6; 1.0	52.5; 46.8	485; 491
				OS: clear			
F4	c.81C>T (p.R27R)	Mother	26	OU: clear	1.0; 1.0	53; 51.8	399; 402
F5	c.81C>T (p.R27R)	Father	17	OU: clear	1.0; 1.0	45.7; 46.2	460; 463
F6	c.425-73C>T	Mother	10	OD: Fleischer’s ring, Vogt’s line	0.3; 1.0	60; 46.3	457; 495
				OS: clear			

OD: right eye; OS: left eye; OU: both eyes.

**FIGURE 4:**
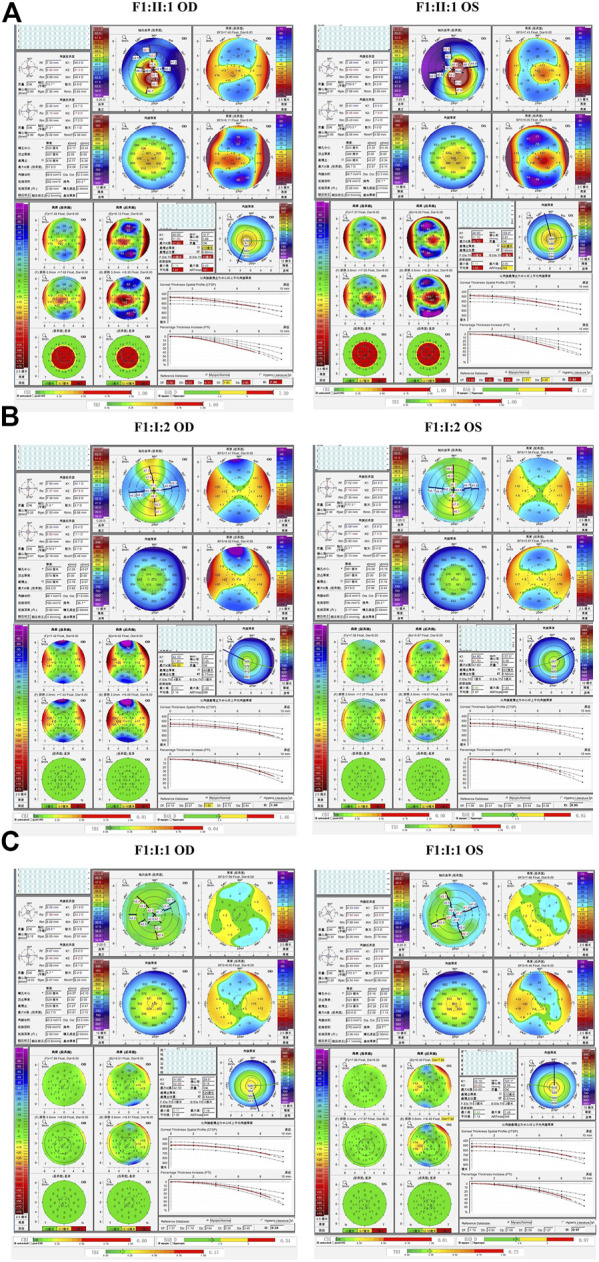
Pentacam and Corvis ST images of the proband and first-degree relatives carrying the p.G342E variant in F1. **(A)** Abnormal maps of the proband. **(B)** Pentacam examination of the mother with the variant revealed one suspicious parameter (Dp) and one abnormal parameter (Kmax) in the right eye, while one suspicious parameter (TBI) was detected by Corvis ST examination of the left eye. **(C)** Pentacam and Corvis ST examinations of the father (F1: I:1) without the c.1025G>A variant revealed normal parameters. OD: right eye; OS: left eye.

In the first family (F1), the *VSX1* variant p.G342E was discovered in both the proband and his mother who had no self-reported symptoms. Slit lamp microscopy revealed bilateral corneal thinning, Fleischer’s ring and Vogt’s line in the cornea of the proband. In his mother, one suspicious parameter (Dp) and one abnormal Kmax was detected in Pentacam examinations of the right eye, as well as one suspicious parameter (TBI) in Corvis ST examination of the left eye (Figure 4).

**FIGURE 5:**
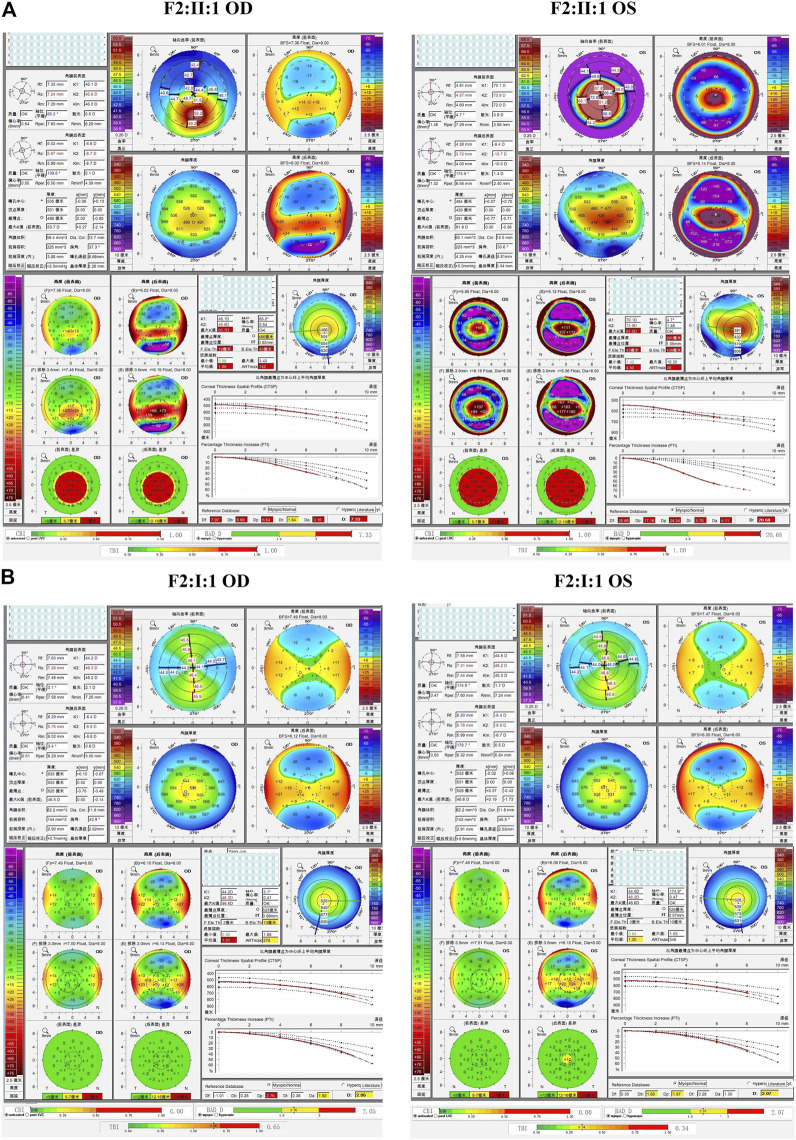
Pentacam and Corvis ST images of the proband and asymptomatic first-degree relatives carrying the p.G160V variant in F2. **(A)** Abnormal corneal topography and biomechanics of the proband. **(B)** Pentacam and Corvis ST examinations of the father with the p.G160V variant revealed five suspicious parameters (ARTmax, B.Ele.Th, Da, BAD-D, and TBI) and one abnormal parameters (Dp) in the right eye, while four suspicious parameters (Db, Dp, BAD-D and TBI) were detected in the left eye.

In the second family (F2), the proband carried the missense variant p.G160V of *VSX1*, which was inherited from the asymptomatic father. Slit lamp microscopy revealed bilateral corneal thinning in both eye and Fleischer’s ring in the left eye of the proband. Pentacam and Corvis ST examinations of his father revealed five suspicious parameters (ARTmax, B.Ele.Th, Da, BAD-D, and TBI) and one abnormal parameters (Dp) in the right eye and four suspicious parameters (Db, Dp, BAD-D and TBI) in the left eye (Figure 5).

**FIGURE 6:**
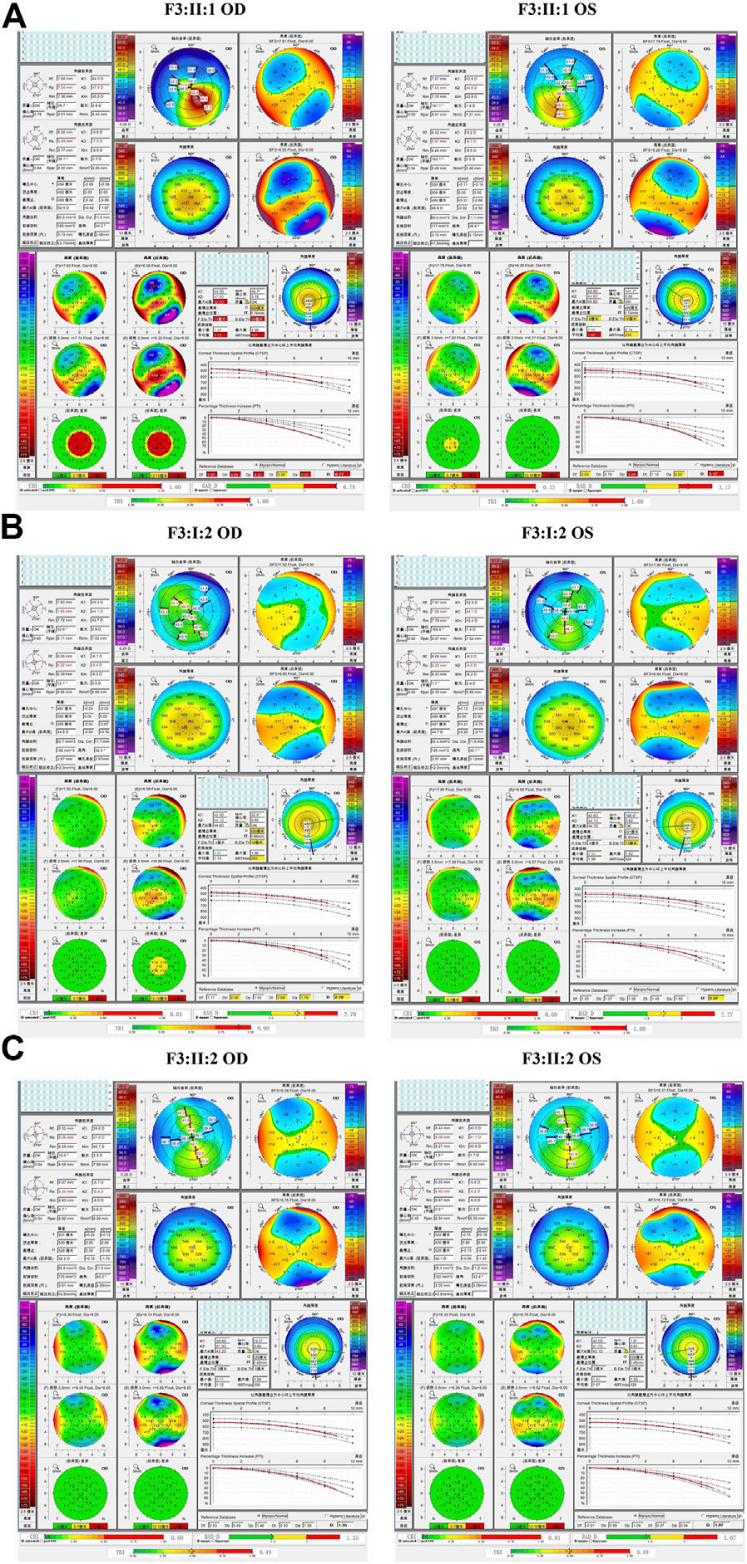
Pentacam and Corvis ST images of the proband and asymptomatic first-degree relatives carrying the p.L17V variant in F3. **(A)** Abnormal corneal topography and biomechanics of the proband. **(B)** The mother with variant was found to have eight suspicious parameters TP, ARTmax, B.Ele.Th, Db, Dt, Da, BAD-D, and TBI in the right eye, while three suspicious parameters B.Ele.Th, BAD-D and TBI in the left eye. **(C)** The brother with variant was identified to have one suspicious parameters TBI in both eyes.

In the third family (F3), the proband (II:1) and her brother (II:2) carried the missense variant p.L17V in *VSX1*, which was inherited from their asymptomatic mother (I:2). Slit lamp microscopy revealed bilateral corneal thinning in both eyes and Fleischer’s ring in the right eye of the proband. Pentacam and Corvis ST examinations of the mother revealed eight suspicious parameters (TP, ARTmax, B.Ele.Th, Db, Dt, Da, BAD-D, and TBI) in the right eye, and three suspicious parameters (B.Ele.Th, BAD-D and TBI) in the left eye. The proband’s 13-year-old asymptomatic brother was found to have one suspicious parameter (TBI) in both eyes (Figure 6).

**FIGURE 7:**
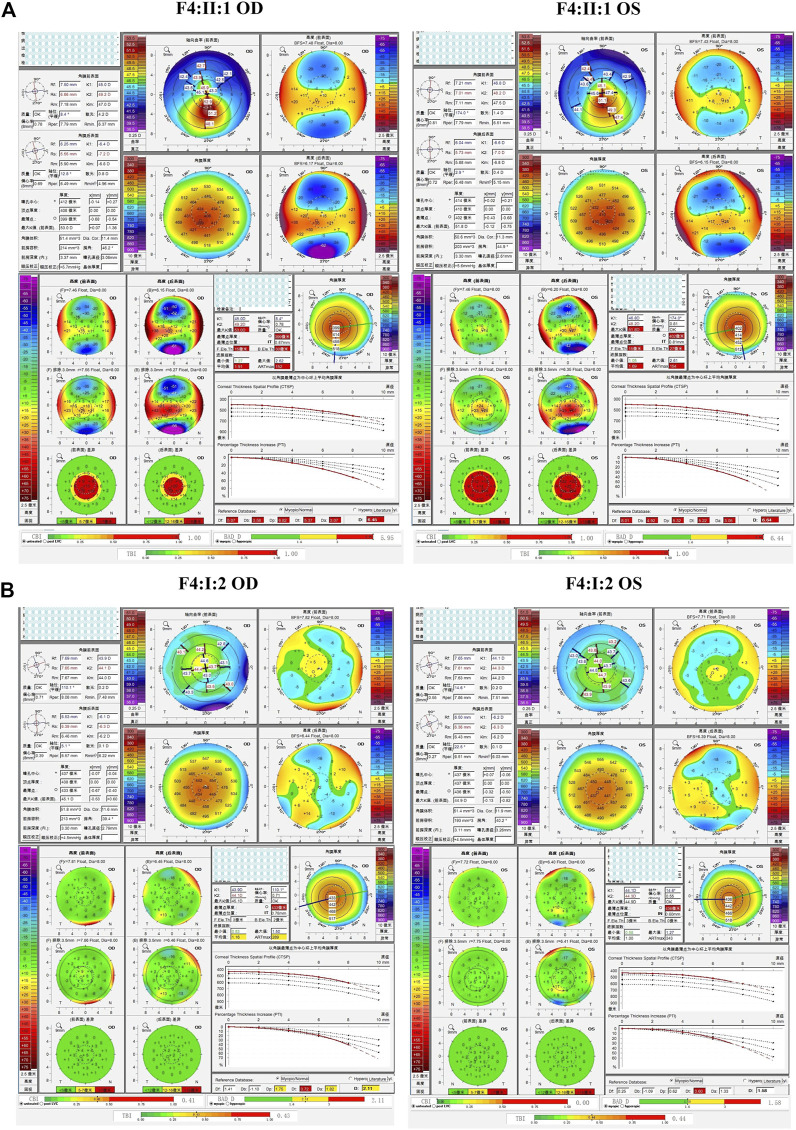
Pentacam and Corvis ST image of the proband and asymptomatic first-degree relatives who carrying the variant p.R27R in family4. **(A)** Abnormal corneal topography and biomechanics of the proband. **(B)** Pentacam and Corvis ST examinations of the mother with the variant revealed six suspicious parameters (ARTmax, BAD-D, Dp, Da, CBI, and TBI) and two abnormal parameters (TP, and Dt) in the right eye, while one suspicious parameter (TBI) and two abnormal parameters (TP, and Dt) were detected in the left eye.

**FIGURE 8:**
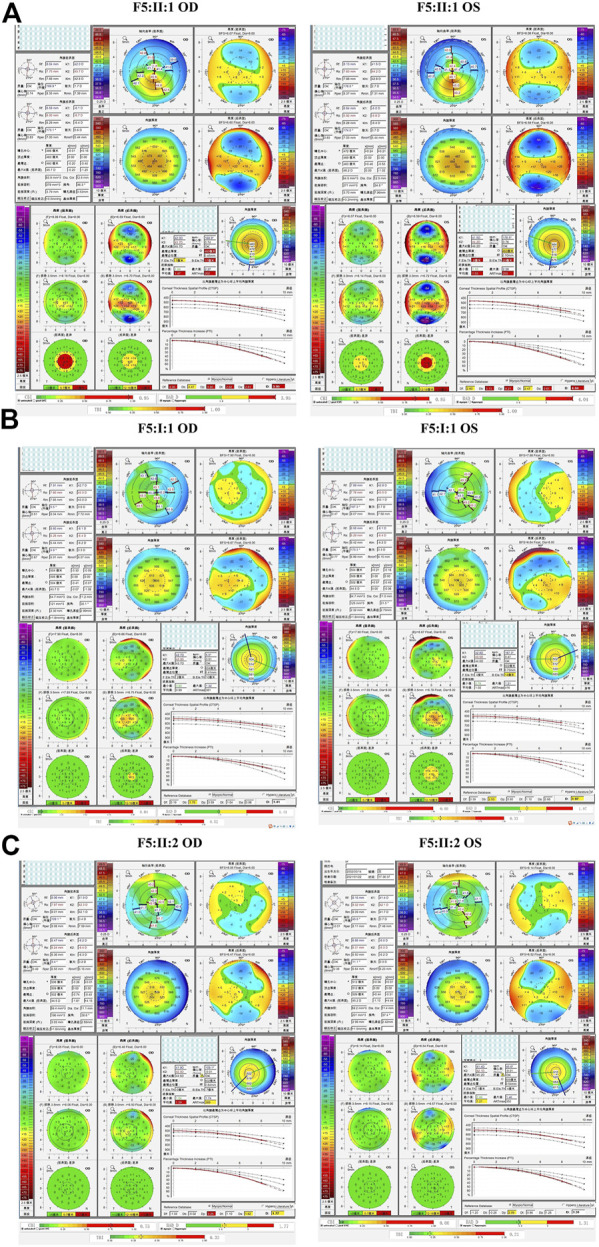
Pentacam and Corvis ST images of the proband and asymptomatic first-degree relatives carrying the p.R27R variant in F5. **(A)** Abnormal corneal topography and biomechanics of the proband. **(B)** Pentacam and Corvis ST examinations of the father with the variant revealed two suspicious parameters (Db and TBI) in the right eye, while four suspicious parameters (B.Ele.Th, Db, BAD-D and TBI) were detected in the left eye. **(C)** Pentacam and Corvis ST examinations of the brother with the variant revealed four suspicious parameters (ARTmax, Da, BAD-D, and TBI) and two abnormal parameters (Dp, and CBI) in the right eye, while one suspicious parameter (Dp) was detected in the left eye.

The fourth and fifth families (F4 and F5) were found to carry the same *VSX1* p.R27R mutation. The probands in both families were characterized by steepness of the inferior cornea in Pentacam. In F4, the variant was revealed in both the proband (II:1) and her mother (I:2) who had no self-reported symptoms. Pentacam and Corvis ST examinations of the mother revealed six suspicious parameters (ARTmax, BAD-D, Dp, Da, CBI, and TBI) and two abnormal parameters (TP, and Dt) in the right eye, while only suspicious parameter (TBI) and two abnormal parameters (TP, and Dt) were detected in the left eye (Figure 7). In F5, the proband (II:1) and his 15-year-old asymptomatic brother (II:2) carried the variant, which was inherited from their asymptomatic father (I:1). Pentacam and Corvis ST examinations of the father revealed two suspicious parameters (Db and TBI) in the right eye, while four suspicious parameters (B.Ele.Th, Db, BAD-D and TBI) were detected in the left eye. Pentacam and Corvis ST examinations of the brother (II:2) revealed four suspicious parameters (ARTmax, Da, BAD-D, and TBI) and two abnormal parameters (Dp, and CBI) in the right eye, while only one suspicious parameter (Dp) was detected in the left eye (Figure 8).

**FIGURE 9:**
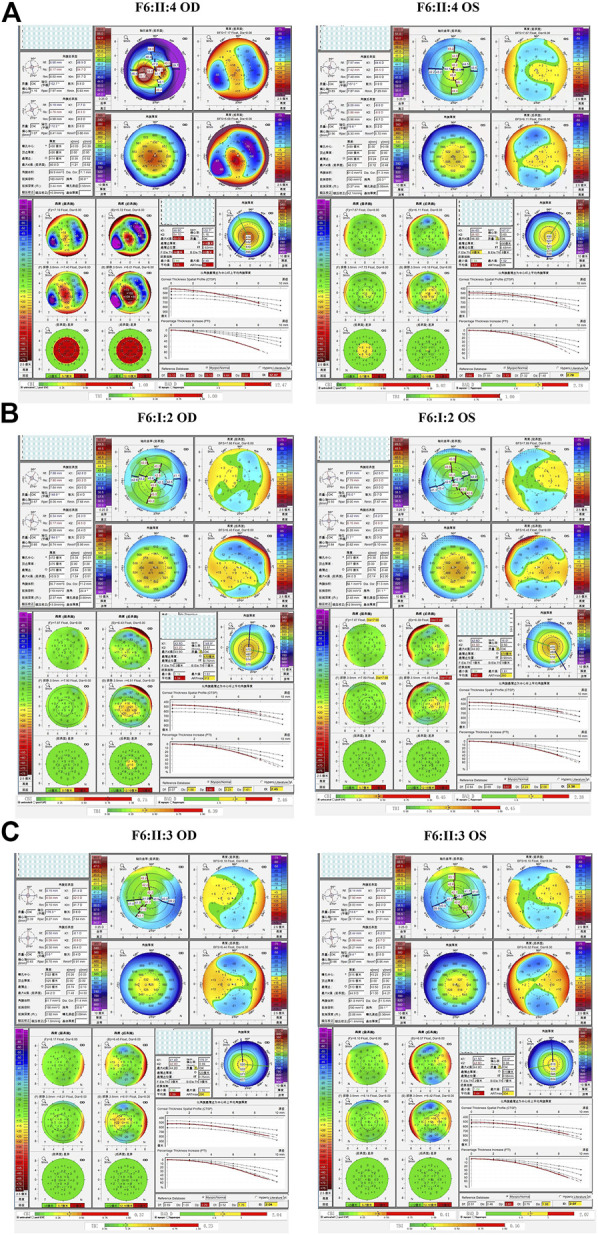
Pentacam and Corvis ST images of the proband and asymptomatic first-degree relatives carrying the c.425-73C>T variant in F6. **(A)** Abnormal corneal topography and biomechanics of the proband. **(B)** Pentacam and Corvis ST examinations of the mother with the variant revealed eight suspicious parameters (TP, ARTmax, Db, Dt, Da, BAD-D, CBI, and TBI) and one abnormal parameter (Dp) in the right eye, while seven suspicious parameters (TP, ARTmax, Dt, Da, BAD-D, CBI, and TBI) and one abnormal parameter (Dp) were detected in the left eye. **(C)** Pentacam and Corvis ST examinations of the sister with the variant revealed have four suspicious parameters (ARTmax, Da, BAD-D, and CBI) and one abnormal parameter (Dp) in both eyes.

In the sixth family (F6), the proband (II:4) and her 12-year-old asymptomatic sister (II:3) carried the c.425-73C>T variant in the first intron of *VSX1*, which was inherited from their asymptomatic mother (I:2). Pentacam and Corvis ST examinations of the mother revealed eight suspicious parameters (TP, ARTmax, Db, Dt, Da, BAD-D, CBI, and TBI) and one abnormal parameter (Dp) in the right eye, while seven suspicious parameters (TP, ARTmax, Dt, Da, BAD-D, CBI, and TBI)and one abnormal parameter (Dp) were detected in the left eye. Pentacam and Corvis ST examinations of the proband’s sister (II:3) revealed four suspicious parameters (ARTmax, Da, BAD-D, and CBI) and one abnormal parameters (Dp) in both eyes (Figure 9). In slit lamp microscopy examinations, Fleischer’s ring and Vogt’s line were visible in the cornea of the proband’s right eye.

**FIGURE 10:**
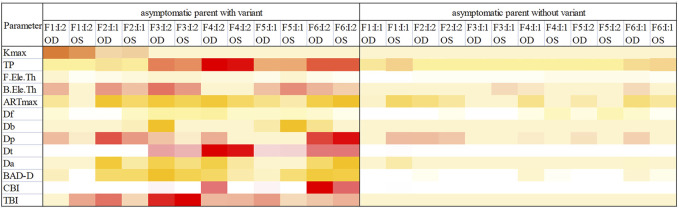
Heatmap of parameters from Pentacam and Corvis ST examinations of the parents in the six families. Darker color means the indicator is more likely to be abnormal. OD, right eye; OS, left eye.

Corneal topographic and Corvis ST changes in ultra-early KC were found in asymptomatic parents, with the abnormal parameters mainly including TP, Db, Dt, Da,B.Ele.Th, TBI (Figure 10).

## 4 Discussion

With the development of next-generation sequencing, many potentially pathogenic mutations of *VSX1* have emerged, including p.A131P, p.G160V, p.L17P, p.D144E, p.L159M, p.G160D, p.R166W, p.H244R and p.P247R, which were identified in several studies on the genetics of KC and screened from the PubMed database (Héon et al., 2002; Bisceglia et al., 2005; Valleix et al., 2006). However, there are few reports of *VSX1*-related sequence variations in China (Guan et al., 2017). In this study, we investigated the potentially pathogenic variants of KC by screening *VSX1* sequence variations and clinical features of affected families from northwest China. Based on a comprehensive analysis of the MAF, conservativeness, pathogenicity prediction, co-segregation and previous reports, our data implicated the variants p.G160V, p.L17V, and p.G342E as the potentially pathogenic variants of KC in this patient population.

The variant p.G342E was identified in a 19-year-old male with a 4-year history of KC that was absent from all the controls. According to the gnomAD database, the MAF of this variant is extremely low (0.00001). The Human *VSX1* gene, which has five exons encoding a 365-amino-acid protein with a homeobox DNA binding domain and a CVC domain, is highly conserved among vertebrates (Guan et al., 2017). Furthermore, homeobox genes characteristically play a dominant role in the regulation of gene expression (Mok et al., 2008). Therefore, the variant p.G342E in the coding region of the exon 5 may lead to regulatory changes in *VSX1* gene expression. Sanger sequencing confirmed that the variant was inherited from the proband’s mother, who showed signs of KC changes on the anterior surface of the cornea in both eyes in the Pentacam and Corvis ST examinations. Thus, it was speculated that the maternally-inherited p.G342E variant is responsible for KC in this pedigree.

In a study of 249 patients with KC in the Korean region in 2008, Mok et al. (2008) revealed that the p.G160V variant of *VSX1* identified in 13 patients (5.3%) increased the risk of KC. In 2017, Guan et al. (2017) identified the p.G160V variant of *VSX1* at a rate of 4.0% in a study of 50 patients with KC in China, but failed to detect the sequence variation in controls, thus implicating this variant as the causative gene of KC in these two patients. Our findings (p.G160V, 2.6%) in this study are consistent with these reports. Furthermore, the p.L17V variant of *VSX1* inherited from the mother may be another disease-causing gene in this case of KC since this variant is highly conserved and predicted to be pathogenic by the Mutation Assessor, FATHMM, MetaLR, and DANN software (DANN score 0.991). However, based on a case-control study (53KC cases and 100 controls) in Korea, Jeoung et al. (2012) concluded that p.G160V and p.L17V were not pathogenic since they identified p.G160V in threeKC cases (5.7%) and three controls (3%) and p.L17V in twoKC cases (3.8%) and one controls (3%). We consider that the discrepancy between these findings and those of our study are due to the phenotypically heterogeneous nature of KC, probably resulting from differences in ethnicity, environment and other factors. Second, since the study by Jeoung was completed in 2012, there were likely to be significant equipment-related challenges to early screening of KC, particularly for patients in the earliest stages of disease development. Hence, it is essentially impossible to rule out the possibility that a patient with early KC was included in the control group.

The synonymous sequence variation p.R27R identified in 37 cases (5.4%) in this study was also reported in five cases (9.4%) in a study of 53 Korean patients with KC (Jeoung et al., 2012). It was proposed that the lack of change in the amino acid at this position in the VSX1 protein indicates that this variation is a single nucleotide polymorphism in the *VSX1* gene. However, it is interesting to note that the clinical phenotypes of the two patients with the p.R27R synonymous sequence variation in this study were very similar (both had BCVA of 1.0 and had no obvious changes in the cornea, which showed only high curvature in topography examination). Shen et al. (2022) studied thousands of coding sequence variations in different yeast genes under four distinct environmental conditions and speculated that synonymous sequence variations are almost as important as non-synonymous sequence variations in causing disease. However, due to the absence of functional verification, we speculated that the synonymous mutation p.R27R, which has been reported at high incidence in previous studies (Jeoung et al., 2012), is likely to be uncertain clinical significance in KC at present.

Furthermore, it is interesting to emphasize that two patients with the p.R27R mutation had a moderate phenotype, while the patient with the p.G342E mutation had a more severe phenotype; thus, we hypothesize that the severity of the KC is associated with different gene sequence variations, with different phenotypic levels corresponding to distinct prognoses. Therefore, screening the genotypes of KC patients may provide valuable information to determine which patients should receive early intervention.

Surprisingly, the clinical examinations of these six families revealed ultra-early KC changes in topographic and biomechanical indicators in the asymptomatic first-degree parents who shared the proband’s sequence variation, indicating that these relatives are at increased risk of developing latent subclinical KC (Figure 10). Although patients with the same *VSX1* variant genotype can exhibit various clinical symptoms, there is still evidence that co-segregation is compatible with autosomal dominant inheritance of the clinical symptoms and genotypes for these *VSX1* variants. Kriszt et al. (2014) found KC to be a complex non-standard Mendelian genetic disorder by studying the results of family co-segregation, an approach that is commonly used by many researchers. The complexity of KC may arise not only from genetic mechanisms, but also from epigenetic influences, such as those regulating the degree of RNA and DNA methylation, and the interaction between different proteins that may also affect epistasis (Eichler et al., 2010). An Italian study of 302KC patients (De Bonis et al., 2011), which is the largest study population reported to date, highlighted the possibility of variable expression and incomplete epistasis of *VSX1* sequence variations in KC. Chen et al. (2021) conducted genetic and clinical studies on 88KC families and reported that relatives were at higher risk of developing latent subclinical KC. In accordance with our study, they also observed variable expression of the clinical phenotype of KC among relatives carrying the same sequence variation. These results are consistent with our findings and suggest the existence of variation in the clinical phenotypes of KC with the same genetic variation. With the rapid global increase in the number of myopic individuals and the recent rise in the popularity of refractive surgery, the risk of postoperative secondary KC has increased in patients without clinical signs of risk factors prior to surgery (Klein et al., 2006). Therefore, it is important to overcome the challenges to accurate recognition of these cases of latent subclinical KC. Indeed, our study indicates that genetic testing may facilitate the diagnosis of latent subclinical KC and re-classification of the disease.

In summary, our study provides evidence of the involvement of the p.G342E, p.L17V variant of *VSX1* in the pathogenesis of KC and they were described for the first time in Chinese KC patients. These results expand the spectrum of *VSX1* sequence variations. Moreover, collectively, our data indicate that the *VSX1* variants identified in both the patients and asymptomatic first-degree relatives may be major genetic predisposing factors that influence KC through an autosomal dominant inheritance pattern with variable expressivity. We propose that genetic screening is a valuable approach to detecting subclinical KC at an extremely early stage, or even identifying latent subclinical KC to avoid corneal ectasia after refractive surgery. However, it should be noted that our study is limited by the small number of KC families included; therefore, further studies in more KC patients are required to validate our findings and clarify the correlation between genotype and phenotype.

## Data Availability

The data presented in the study are publicly available. This data can be found here: http://www.ncbi.nlm.nih.gov/bioproject/941955.
